# Adiposity Is Associated with a Higher Risk of Thyroid Malignancy in Patients with Hashimoto’s Thyroiditis

**DOI:** 10.3390/diagnostics15070853

**Published:** 2025-03-27

**Authors:** Maria Fokou, Aliki Economides, Elpida Demetriou, Demetris Lamnisos, Aris P. Agouridis, Panagiotis Papageorgis, Panayiotis A. Economides

**Affiliations:** 1 Department of Medicine, School of Medicine, European University Cyprus, Nicosia 2404, Cyprus; mf162105@students.euc.ac.cy (M.F.); ed181861@students.euc.ac.cy (E.D.); a.angouridis@euc.ac.cy (A.P.A.); 2 Department of Health Sciences, European University Cyprus, Nicosia 2404, Cyprus; aeconomi@cytanet.com.cy (A.E.); d.lamnisos@euc.ac.cy (D.L.); 3 Economides Thyroid & Endocrinology Center, Nicosia 2406, Cyprus; 4 Department of Life Sciences, European University Cyprus, Nicosia 2404, Cyprus; p.papageorgis@euc.ac.cy

**Keywords:** hashimoto’s thyroiditis, HT, thyroid nodules, thyroid cancer, obesity, body mass index, BMI

## Abstract

**Background/Objectives**: Both adiposity and Hashimoto’s thyroiditis (HT) are states of chronic inflammation. Adiposity may increase the risk of thyroid nodules and thyroid carcinoma. However, its role in patients with HT remains unclear. The connections among thyroid nodularity, adiposity, and HT have not been explored. **Aim**: To investigate the impact of adiposity on thyroid nodularity in patients with HΤ and to determine whether there are any differences in the risk for thyroid cancer. **Methods**: This retrospective cohort study included 294 consecutive patients with HT who were categorized according to their body mass index (BMI). Grayscale ultrasound (US) and fine-needle aspiration (FNA) cytology results were evaluated in association with clinicopathological characteristics. **Results**: After controlling for age and gender, nodules from patients with a BMI ≥ 25 kg/m^2^ showed significantly more suspicious or malignant cytology (Thy 4–5) compared to patients with a BMI < 25 kg/m^2^ (27.03% vs. 18.18%; *p* < 0.01). Although not statistically significant after adjustments, patients with BMI ≥ 25 kg/m^2^ demonstrated a higher proportion of nodules classified as highly suspicious on ultrasound (28.20% vs. 22.46%). Additionally, overweight and obese patients tended to have more thyroid nodules (mean ± SD: 2.91 ± 2.11) than normal-weight patients (2.36 ± 1.79), a difference approaching marginal significance (*p* = 0.06). **Conclusions**: Adiposity is associated with more suspicious and malignant cytology in patients with HT. Overweight and obese patients with HT tended to have more thyroid nodules. Further studies are needed to investigate the mechanisms linking obesity, thyroid nodules, and HT.

## 1. Introduction

Hashimoto’s thyroiditis (HT) is considered the most common type of autoimmune thyroid disorder and the most common cause of hypothyroidism [1,2,3]. Clinically, it is characterized by the loss of immunological tolerance against the thyroid gland, lymphocytic infiltration, and the presence of thyroid autoantibodies [4,5]. The specific etiology of the disease is not fully understood, although it includes multiple factors such as genetic, environmental, nutritional, sex, parenthood, and age [6,7,8,9].

Numerous studies have suggested a possible association between HT and thyroid cancer [10,11,12]. The inflammatory process of HT, such as lymphocytic infiltration and tissue fibrosis, can cause significant structural changes in the thyroid gland, increasing the suspicion of cancer based on ultrasonographic results [13,14]. However, the role of HT in the risk of developing thyroid cancer remains debatable [11,12]. Papillary thyroid carcinoma (PTC) is more frequently observed in nodular HT than in nodular goiter and is associated with high levels of thyroid-stimulating hormone (TSH) [15].

Obesity is a condition in which the accumulation of fat increases and is determined by the body mass index (BMI), the most practical method for the clinical screening of the population for obesity [16,17,18,19]. According to the World Health Organization (WHO), 43% of adults were overweight and 16% were obese in 2022 [20]. Although certain studies have reported an increased risk of thyroid nodules in individuals with a higher BMI, others have not reported a direct association [21,22,23,24,25,26,27,28]. The connections among thyroid nodularity, obesity, and HT have not been explored in the literature. In a recent study by our group [29], we demonstrated that increased adiposity is associated with a higher prevalence of thyroid nodules and worse fine-needle aspiration (FNA) outcomes in nodules of patients without HT. Our current research further investigates the impact of adiposity on thyroid malignancy risk, specifically in patients with HT. In our study, we hypothesized a link between obesity, thyroid nodules, and HT, potentially mediated via the chronic inflammation observed in both conditions. Our study aimed to investigate the relationships among obesity, thyroid nodules, and HT.

The possible association between obesity and thyroid nodularity has been investigated with mixed results. A community-based study in China revealed a positive association between thyroid nodularity and obesity; however, waist circumference had a stronger association, emphasizing central adiposity as a key factor [30]. The same association was observed in a cross-sectional study in Italy, mainly in females and older individuals, with obese patients presenting larger nodules than their non-obese counterparts [31]. Panagiotou et al. highlighted a greater prevalence of thyroid nodules in overweight and obese individuals, where age and the female sex were independent predictors [32]. The same positive association was observed between the risk of thyroid nodules and obesity in a large study of adults and children involving 9203 subjects [33]. A pediatric study revealed a positive association of obesity based on BMI, body surface area (BSA), waist circumference, and thyroid nodules. Girls demonstrated a higher likelihood of having multiple nodules, whereas boys presented with more solitary nodules [34]. In another study with patients up to 21 years of age, a higher BMI was associated with an elevated risk of malignant thyroid nodules [35].

Conversely, a retrospective study of 4849 patients demonstrated a negative association between obesity and thyroid nodules, suggesting that severe obesity may have a protective effect against thyroid cancer [27]. Layegh et al. reported no significant association between thyroid nodules and obesity; however, they noted a higher prevalence of thyroid nodules among participants with insulin resistance. In this study, thyroid volume was more closely associated with BMI [28]. Another study regarding morbidly obese women reported a potentially inverse relationship between extreme obesity and thyroid nodules [36]. The inconsistent findings regarding the association between obesity and thyroid nodularity can be attributed to differences in the study designs, study population characteristics, measurement methods, genetic predispositions, and variations in imaging techniques.

Obesity is characterized by chronic low-grade inflammation, marked by increased proinflammatory cytokines and decreased anti-inflammatory adipokines, which activate key pathways such as Nuclear Factor kappa-light-chain-enhancer of activated B cells (NF-κB), Signal Transducer and Activator of Transcription 3 (STAT3), and Activator Protein 1 (AP-1). These pathways promote tumor cell proliferation, angiogenesis, and oxidative stress, contributing to the development of thyroid tumors [37]. Chronic inflammation is also present in Hashimoto’s thyroiditis, an autoimmune thyroid disorder characterized by tissue damage, fibrosis, and the release of inflammatory cytokines including Interleukin 6 (IL-6), Tumor Necrosis Factor-alpha (TNF-α), and Interleukin 1 beta (IL-1β) [38,39]. This inflammatory and oxidative microenvironment presumably leads to DNA damage, genomic instability, neoplastic transformation, and increased risk of PTC.

## 2. Materials and Methods

### 2.1. Patients and Data Recording

This was a retrospective cohort study of 294 consecutive patients who were diagnosed with both Hashimoto’s thyroiditis and thyroid nodules (solitary or multiple) over two years. The inclusion criteria were the diagnosis of both thyroid nodules (solitary or multiple) and HT. Patients with no nodules and pregnant or pediatric patients were excluded.

All the patients completed their medical, endocrine, and ultrasonographic evaluations at the Thyroid & Endocrinology Center, a referral clinic and a teaching affiliate of the European University Cyprus School of Medicine. HT was diagnosed based on the clinical presentation, medical history, US findings, and the presence of thyroid peroxidase (TPO) and thyroglobulin (Tg) antibodies. All the patients underwent thyroid and neck ultrasound (US) examination by a single experienced endocrinologist via a GE Logiq E9 system with an ML 6-15 probe, ensuring consistency in image acquisition and interpretation, thereby minimizing inter-operator variability [40,41]. The ultrasonographic findings were mapped onto a detailed diagram, indicating the location and dimensions of the thyroid nodules. A thyroid nodule was considered as any discrete lesion compared to the surrounding thyroid parenchyma measuring a minimum of 0.2 cm. Inflammatory or pseudo nodules were excluded from the analysis. The ultrasound malignancy risk was recorded, and nodules underwent ultrasound-guided fine-needle aspiration (FNA) according to the American Thyroid Association (ATA) 2015 guidelines [42].

The data recorded included sex, age, weight, height, TSH level, number of nodules, and maximal diameter of the largest nodule. The BMI was calculated using the World Health Organization (WHO) classification by measuring the weight and height of the patient at the initial evaluation appointment. The two groups were categorized based on the BMI: normal (BMI < 25 kg/m^2^) and overweight with obesity (BMI ≥ 25 kg/m^2^), as this threshold is commonly used in clinical and research settings to assess metabolic risk factors. The cytological diagnosis was categorized based on the reporting of the Royal College of Pathologists [43]. The Cyprus National Bioethics Committee approved this study (ΕΕΒΚ ΕΠ 2022.01.88). The personal information and identities of patients were maintained fully confidential throughout the study.

### 2.2. Statistical Analysis

The following parameters were examined according to the BMI groups: age, sex, TSH levels, number of nodules, maximal nodular diameter, ultrasound malignancy risk, and FNA cytology results. The collected data were presented as the means and standard deviations (SD) for numerical variables, whereas categorical variables were presented as the absolute frequencies and percentages. A comparison of continuous variables between the two BMI groups was performed via an independent samples *t*-test. For the categorical variables, sex, ultrasonographic malignancy risk, and FNA results, a chi-square test was performed. A two-sided value of *p* < 0.05 was considered statistically significant. The data were entered into an Excel worksheet, and a statistical analysis was performed via the IBM-SPSS 20 statistical analysis software package. There were no missing data in the variables analyzed in this study.

A post hoc power analysis was conducted to evaluate the statistical power of the observed comparison between the two BMI groups (BMI ≥ 25 kg/m^2^, *n* = 156; BMI < 25 kg/m^2^, *n* = 138) with respect to the number of thyroid nodules. The analysis was performed using G*Power 3.1.9.7 based on the observed effect size (Cohen’s d) derived from the independent samples *t*-test results. The mean number of thyroid nodules was significantly higher in the BMI ≥ 25 kg/m^2^ group (2.91 ± 2.11) compared to the BMI < 25 kg/m^2^ group (2.36 ± 1.79), with a *p*-value of 0.02. The calculated effect size (Cohen’s d) was 0.29, indicating a small to medium effect. With the given sample sizes (n_1_ = 156, n_2_ = 138), a significance level (α) of 0.05, and the observed effect size, the post hoc power analysis revealed a statistical power of 0.72 (72%). This suggests that the study had a moderate likelihood of detecting a true difference in the number of thyroid nodules between the two BMI groups. While the power is below the conventional threshold of 0.80, the significant *p*-value (0.02) supports the robustness of the observed findings. Future studies with larger sample sizes may be warranted to further confirm these results with higher statistical power.

To account for potential confounding factors, gender, and age, multivariable regression models were applied. Specifically, multiple binary or ordinal logistic regression was used for binary and ordinal categorical outcomes, multiple linear regression was applied for continuous variables, and multiple Poisson regression was employed for count data.

## 3. Results

### 3.1. Clinicopathological Characteristics (Table 1)

A total of 294 patients with thyroid nodules were classified into two groups based on their BMI: Group 1 (BMI < 25, *n* = 138) and Group 2 (BMI ≥ 25, *n* = 156). Gender distribution differed significantly between groups, with males comprising a higher proportion in Group 2 (19.23%) compared to Group 1 (7.97%; *p* = 0.01). After adjusting for gender and age, this difference remained significant (*p* = 0.03). Additionally, the patients in Group 2 were significantly older (mean age 50.47 ± 14.82 years) compared to Group 1 (mean age 42.43 ± 13.28 years, *p* < 0.001), an association that persisted after adjusting for gender and age (*p* < 0.001).

**Table 1:** Clinicopathological characteristics according to BMI.

Clinical Characteristics	Group 1 BMI < 25 (*n* = 138)	Group 2 BMI ≥ 25 (*n* = 156)	*p*-Value	*p*-Value
Gender				
Male (*n*, %)	11 (7.97%)	30 (19.23%)	0.01 ^†^	0.03 ^§^
Female (*n*, %)	127 (92.03%)	126 (80.77%)		
Age (years, mean ± SD)	42.43 (13.28)	50.47 (14.82)	0.00 *	0.00 ^¶^
TSH levels (mU/L)				
Mean ± SD	3.92 (10.73)	3.31 (6.37)	0.55 *	0.77 ^¶^
Solitary nodule	52 (37.68%)	41 (26.28%)	0.048 ^†^	0.08 ^§^
Multiple nodules	86 (62.32%)	115 (73.72%)		
No of nodules				
Mean ± SD	2.36 (1.79)	2.91 (2.11)	0.02 *	0.06 ^‡^
Maximal nodular diameter (cm)				
Mean ± SD	1.26 (1.12)	1.18 (0.85)	0.47 *	0.16 ^¶^
2015 ATA				
Very low to low suspicion	66 (47.83%)	66 (42.31%)	0.49 ^†^	0.18 ^±^
Intermediate suspicion	41 (29.71%)	46 (29.49%)		
High suspicion	31 (22.46%)	44 (28.20%)		

Continuous variables were expressed as mean ± SD; categorical variables were expressed as frequency and percentages. * independent samples *t*-test. ^†^ chi-square test. ^§^ multiple logistic regression adjusting for gender and age. ^¶^ multiple linear regression adjusting for gender and age. ^‡^ multiple Poisson regression adjusting for gender and age. ± multiple ordinal logistic regression adjusting for gender and age.

There was no significant difference in the serum TSH levels between the two groups (3.92 ± 10.73 mU/L vs. 3.31 ± 6.37 mU/L, *p* = 0.55), and this remained non-significant after adjustment (*p* = 0.77). Multiple nodules were more prevalent in Group 2 (73.72%) compared to Group 1 (62.32%). The mean number of nodules was significantly greater in patients with BMI ≥ 25 (2.91 ± 2.11) compared to those with BMI < 25 (2.36 ± 1.79, *p* = 0.02), though this association was attenuated after adjustment (*p* = 0.06).

Maximal nodular diameter did not differ between the groups (1.26 ± 1.12 cm in Group 1 vs. 1.18 ± 0.85 cm in Group 2, *p* = 0.47), and adjustment for age and gender confirmed no significant relationship (*p* = 0.16). No significant differences were observed between the groups in the distribution of very low to low suspicion, intermediate suspicion, or high suspicion nodules, even after adjustment for gender and age (*p* = 0.18).

### 3.2. FNA Cytology Results (Table 2)

Fine-needle aspiration (FNA) cytology was performed on 140 nodules (Group 1, *n* = 66; Group 2, *n* = 74). The cytological findings were categorized as Thy 2 (benign), Thy 3 (indeterminate), and Thy 4–5 (suspected malignancy/malignant). Although initial unadjusted analysis showed no significant difference in cytological categories (*p* = 0.13), adjusted analysis for gender and age revealed a significant association between higher BMI and higher-risk cytology categories (*p* < 0.01). Specifically, Group 2 demonstrated a higher prevalence of Thy 3 (10.81%) and Thy 4-5 nodules (27.03%) compared to Group 1 (4.55% and 18.18%, respectively). The proportion of benign (Thy 2) nodules was higher in Group 1 (77.27%) compared to Group 2 (62.16%).

**Table 2:** FNA results (*n* = 140).

FNA Results	Group 1 BMI < 25 (*n* = 66)	Group 2 BMI ≥ 25 (*n* = 74)	*p*-Value	*p*-Value
Thy 2	51 (77.27%)	46 (62.16%)	0.13 ^†^	<0.01 ^§^
Thy 3	3 (4.55%)	8 (10.81%)		
Thy 4–5	12 (18.18%)	20 (27.03%)		

Categorical variables were expressed as frequencies and percentages. ^†^ chi-square test. ^§^ multiple ordinal logistic regression adjusting for gender and age.

## 4. Discussion

Considering the alarming number of obesity cases and the increased prevalence of thyroid nodules and HT, exploring the possible relationships among these factors is crucial. The current study showed a significant association between increased BMI and suspicious or malignant cytology in thyroid nodules among patients with HT independent of age and gender. This suggests adiposity could contribute to an increased malignancy risk in thyroid nodules. Although trends were observed towards more frequent highly suspicious ultrasonographic patterns and a higher number of nodules in overweight and obese patients, these did not reach statistical significance.

Fat accumulation is linked with elevated TSH levels [38,44,45,46], and excess adiposity can affect thyroid autoimmunity by increasing the levels of adipokines such as leptin and IL6, which are crucial for maintaining immune homeostasis [39,47,48]. The increased TSH secretion can result from the high level of leptin produced by the adipose tissue, which induces adipocyte proliferation via the TSH receptor at this level [49,50]. Leptin has immunomodulatory functions and when associated with obesity, it can provoke a systemic inflammatory state that can promote the development of autoimmune diseases [51].

In our study, overweight and obese patients had more thyroid nodules than patients with a normal weight, but this difference approached marginal significance. Several studies indicate a connection between obesity and thyroid nodules [52,53]. Xu et al. reported a correlation between BMI and an increased risk of thyroid nodules. Moreover, overweight individuals, particularly those with greater central obesity, demonstrated a significantly higher prevalence of multiple nodules than solitary thyroid nodules [54]. Hu et al. linked thyroid nodules to a higher BMI and other components of the metabolic syndrome, such as insulin resistance. Additionally, there is an age-related increase in the prevalence of thyroid nodules, which is significantly greater in women than in men [55]. Moon et al. reported a link between BMI and an increased incidence of thyroid nodules [56].

Our results demonstrated a non-statistically significant trend for more nodules with highly suspicious sonographic patterns in overweight and obese patients. An elevated risk of thyroid nodules with highly suspicious sonographic patterns was associated with a higher BMI [53]. Chen Y et al. proposed a potential link between obesity and the presence of taller-than-wide nodules [57]. Another study revealed that individuals with severe obesity displayed increased hypoechogenicity and a greater frequency of thyroid nodules during ultrasound examinations. However, no significant distinctions were noted based on the ATA-2015 and Thyroid Imaging Reporting and Data System (Ti-Rads) criteria [58]. Zhao et al. reported that overweight and obese individuals had a greater likelihood of multifocality than non-overweight patients [59].

In our study, overweight and obese patients demonstrated a more suspicious and malignant cytology. Several studies have suggested that HT is associated with thyroid cancer [60,61,62]. A correlation between inflammation and neoplastic changes has been proposed [60,61,63]. Peterson et al. and Son et al. reported a positive relationship between BMI and thyroid cancer among both sexes [64,65]. Jankovic et al. reported no correlation between HT and PTC based on eight fine-needle aspiration studies [66]. Shi et al. reported significantly elevated levels of inflammatory cytokines, including IL-6, TNF-α, and IL-1β, in PTC tissues from patients with coexisting HT than patients with PTC alone. This finding suggests that the chronic inflammatory microenvironment in HT may contribute to DNA damage, oxidative stress, and genomic instability, promoting neoplastic transformation and tumor progression [67]. Similar results were also reported by Zivancevic-Simonovic et al., who reported that PTC patients with HT produced significantly higher concentrations of Interleukin 4 (IL-4), IL-6, Interleukin 9 (IL-9), Interleukin 13 (IL-13), and Interferon-gamma (IFN-γ) than PTC patients without HT [68]. The pathophysiology of thyroid cancer is complex and involves multiple biological mechanisms, and obesity plays a role in this interplay via low-grade inflammation and hormonal and metabolic dysfunction [37]. HT further adds to this interplay owing to its possible association with an increased risk of thyroid nodularity and thyroid cancer.

The main limitation of our study is its single-center retrospective nature. Another limitation is the use of BMI to assess the obesity levels in our population. Although BMI is an effective and reliable measure of the total body mass, it does not indicate the distribution of adiposity. In our study, we did not examine other confounding factors such as the duration of obesity, weight history, family history, prior radiation exposure, medication use, or genetic or environmental differences between the two groups. Future studies should address these limitations through prospective designs with comprehensive data collection on metabolic, genetic, and environmental risk factors.

## 5. Conclusions and Future Perspectives

Our study showed a significant association between increased BMI and suspicious or malignant cytology in the thyroid nodules of patients with HT. Although not statistically significant, overweight and obese patients were more likely to have a higher number of nodules and more nodules, with high suspicion sonographic patterns. Further studies are required to clarify the mechanisms underlying the association of adiposity with thyroid nodules, especially in patients with HT. Future research should focus on preventative weight loss strategies to reduce the incidence of thyroid nodules and cancer development in this population. A clear understanding of the aforementioned possible relationships and mechanisms linking obesity, thyroid nodularity, and HT will enable clinicians to identify at-risk patients and develop screening strategies that can lead to early detection and timely intervention. Considering that obesity is a modifiable risk factor, public health initiatives addressing healthy eating, exercise, healthier lifestyles, and weight loss programs can improve patient management and outcomes. Prospective longitudinal studies with larger sample sizes, genetic and molecular markers, and detailed metabolic profiling, including insulin resistance markers, inflammatory cytokines, and adipokine levels, could provide deeper insights into these relationships. Our findings suggest that clinicians should consider BMI as an independent factor in the risk stratification of thyroid nodules in HT patients. Overweight and obese patients with HT may warrant closer surveillance and a lower threshold for FNA. Future guidelines should consider integrating metabolic risk factors into the existing thyroid nodule risk stratification systems.

## Data Availability

The data supporting the findings of this study are available upon request from the corresponding author, P.A.E.

## References

[1] Caturegli P., De Remigis A., Rose N.R. (2014). Hashimoto Thyroiditis: Clinical and Diagnostic Criteria. Autoimmun. Rev..

[2] Ragusa F., Fallahi P., Elia G., Gonnella D., Paparo S.R., Giusti C., Churilov L.P., Ferrari S.M., Antonelli A. (2019). Hashimotos’ Thyroiditis: Epidemiology, Pathogenesis, Clinic and Therapy. Best Pract. Res. Clin. Endocrinol. Metab..

[3] Kaur J., Jialal I. (2025). Hashimoto Thyroiditis. StatPearls [Internet].

[4] Ferrari S.M., Ragusa F., Elia G., Paparo S.R., Mazzi V., Baldini E., Benvenga S., Antonelli A., Fallahi P. (2021). Precision Medicine in Autoimmune Thyroiditis and Hypothyroidism. Front. Pharmacol..

[5] Franco J.-S., Amaya-Amaya J., Anaya J.-M. (2013). Thyroid Disease and Autoimmune Diseases. Autoimmunity: From Bench to Bedside [Internet].

[6] Ihnatowicz P., Drywien M., Wator P., Wojsiat J. (2020). The Importance of Nutritional Factors and Dietary Management of Hashimoto’s Thyroiditis. Ann. Agric. Environ. Med..

[7] Effraimidis G., Wiersinga W.M. (2014). Mechanisms in Endocrinology: Autoimmune Thyroid Disease: Old and New Players. Eur. J. Endocrinol..

[8] Dong X., Li Y., Xie J., Li L., Wan Z., Kang Y., Luo Y., Wang J., Duan Y., Ding S. (2022). The Prevalence of Thyroid Nodules and Its Factors among Chinese Adult Women: A Cross-Sectional Study. Front. Endocrinol..

[9] Hiromatsu Y., Satoh H., Amino N. (2013). Hashimoto’s Thyroiditis: History and Future Outlook. Hormones.

[10] Lima P.C.M., Moura Neto A., Tambascia M.A., Zantut Wittmann D.E. (2013). Risk Factors Associated with Benign and Malignant Thyroid Nodules in Autoimmune Thyroid Diseases. ISRN Endocrinol..

[11] De Morais N.S., Stuart J., Guan H., Wang Z., Cibas E.S., Frates M.C., Benson C.B., Cho N.L., Nehs M.A., Alexander C.A. (2019). The Impact of Hashimoto Thyroiditis on Thyroid Nodule Cytology and Risk of Thyroid Cancer. J. Endocr. Soc..

[12] Boi F., Pani F., Mariotti S. (2017). Thyroid Autoimmunity and Thyroid Cancer: Review Focused on Cytological Studies. Eur. Thyroid. J..

[13] Omidan N., Zahir S.T., Fateh A. (2019). Cytological and Pathological Evaluation of Hashimoto’s Thyroiditis. Maedica.

[14] de Alcântara-Jones D.M., de Alcântara-Nunes T.F., de Rocha B.O., de Oliveira R.D., Santana A.C.P., de Alcântara F.T., de Faria T.M., da Silva I.C., Araújo L.M.B. (2015). Is There Any Association between Hashimoto’s Thyroiditis and Thyroid Cancer? A Retrospective Data Analysis. Radiol. Bras..

[15] Antonelli A., Ferrari S.M., Corrado A., Di Domenicantonio A., Fallahi P. (2015). Autoimmune Thyroid Disorders. Autoimmun. Rev..

[16] Cetin D., Lessig B.A., Nasr E. (2016). Comprehensive Evaluation for Obesity: Beyond Body Mass Index. J. Am. Osteopath. Assoc..

[17] Arroyo-Johnson C., Mincey K.D. (2016). Obesity Epidemiology Trends by Race/Ethnicity, Gender, and education: National Health Interview Survey, 1997–2012. Gastroenterol. Clin. N. Am..

[18] Schwartz M.W., Seeley R.J., Zeltser L.M., Drewnowski A., Ravussin E., Redman L.M., Leibel R.L. (2017). Obesity Pathogenesis: An Endocrine Society Scientific Statement. Endocr. Rev..

[19] Tremmel M., Gerdtham U.G., Nilsson P.M., Saha S. (2017). Economic Burden of Obesity: A Systematic Literature Review. Int. J. Environ. Res. Public Health.

[20] World Health Organization (2024). Obesity and Overweight.

[21] Shin J., Kim M.H., Yoon K.H., Kang M.I., Cha B.Y., Lim D.J. (2016). Relationship between Metabolic Syndrome and Thyroid Nodules in Healthy Koreans. Korean J. Intern. Med..

[22] Liang Q., Yu S., Chen S., Yang Y., Li S., Hu C., Huang D., Kuang L., Li D. (2020). Association of Changes in Metabolic Syndrome Status With the Incidence of Thyroid Nodules: A Prospective Study in Chinese Adults. Front. Endocrinol..

[23] de Siqueira R.A., Noll M., Rodrigues A.P.d.S., Silveira E.A. (2019). Factors Associated with the Occurrence of Thyroid Nodules in Severely Obese Patients: A Case-Control Study. Asian Pac. J. Cancer Prev..

[24] Sari R., Balci M.K., Altunbas H., Karayalcin U. (2003). The Effect of Body Weight and Weight Loss on Thyroid Volume and Function in Obese Women. Clin. Endocrinol..

[25] Liu Y., Lin Z., Sheng C., Zhu Y., Huang Y., Zhong N., Jia Z., Qu S. (2017). The Prevalence of Thyroid Nodules in Northwest China and Its Correlation with Metabolic Parameters and Uric Acid. Oncotarget.

[26] Li Z., Zhang L., Huang Y., Yang P., Xu W., Faustini-Fustini M. (2019). A Mechanism Exploration of Metabolic Syndrome Causing Nodular Thyroid Disease. Int. J. Endocrinol..

[27] Rotondi M., Castagna M.G., Cappelli C., Ciuoli C., Coperchini F., Chiofalo F., Maino F., Palmitesta P., Chiovato L., Pacini F. (2016). Obesity Does Not Modify the Risk of Differentiated Thyroid Cancer in a Cytological Series of Thyroid Nodules. Eur. Thyroid. J..

[28] Layegh P., Asadi A., Jangjoo A., Layegh P., Nematy M., Salehi M., Shamsian A., Ranjbar G. (2020). Comparison of Thyroid Volume, TSH, Free T4 and the Prevalence of Thyroid Nodules in Obese and Non-Obese Subjects and Correlation of These Parameters with Insulin Resistance Status. Caspian J. Intern. Med..

[29] Demetriou E., Economides A., Fokou M., Lamnisos D., Paschou S.A., Papageorgis P., Economides P.A. (2025). Adiposity Is Associated with a Higher Number of Thyroid Nodules and Worse Fine-Needle Aspiration Outcomes. Eur. Thyroid. J..

[30] Song B., Zuo Z., Tan J., Guo J., Teng W., Lu Y., Liu C. (2018). Association of Thyroid Nodules with Adiposity: A Community-Based Cross-Sectional Study in China. BMC Endocr. Disord..

[31] Buscemi S., Massenti F.M., Vasto S., Galvano F., Buscemi C., Corleo D., Barile A.M., Rosafio G., Rini N., Giordano C. (2018). Association of Obesity and Diabetes with Thyroid Nodules. Endocrine.

[32] Panagiotou G., Komninou D., Anagnostis P., Linardos G., Karoglou E., Somali M., Duntas L., Kita M., Tziomalos K., Pazaitou-Panayiotou K. (2017). Association between Lifestyle and Anthropometric Parameters and Thyroid Nodule Features. Endocrine.

[33] Xu W., Chen Z., Li N., Liu H., Huo L., Huang Y., Jin X., Deng J., Zhu S., Zhang S. (2015). Relationship of Anthropometric Measurements to Thyroid Nodules in a Chinese Population. BMJ Open.

[34] Wang N., Fang H., Fu C., Huang P., Su M., Jiang F., Zhao Q., Chen Y., Jiang Q. (2017). Associations of Adiposity Measurements with Thyroid Nodules in Chinese Children Living in Iodine-Sufficient Areas: An Observational Study. BMJ Open.

[35] Ortega C.A., Gallant J.N., Chen S.C., Ye F., Wang H., Belcher R.H., Weiss V.L. (2021). Evaluation of Thyroid Nodule Malignant Neoplasms and Obesity among Children and Young Adults. JAMA. Netw. Open.

[36] Cappelli C., Pirola I., Mittempergher F., De Martino E., Casella C., Agosti B., Nascimbeni R., Formenti A., Rosei E.A., Castellano M. (2012). Morbid Obesity in Women Is Associated to a Lower Prevalence of Thyroid Nodules. Obes. Surg..

[37] Demetriou E., Fokou M., Frangos S., Papageorgis P., Economides P.A., Economides A. (2023). Thyroid Nodules and Obesity. Life.

[38] Sanyal D., Raychaudhuri M. (2016). Hypothyroidism and Obesity: An Intriguing Link. Indian J. Endocrinol. Metab..

[39] Song R.H., Wang B., Yao Q.M., Li Q., Jia X., Zhang J.A. (2019). The Impact of Obesity on Thyroid Autoimmunity and Dysfunction: A Systematic Review and Meta-Analysis. Front. Immunol..

[40] Hadjisavva I.S., Dina R., Talias M.A., Economides P.A. (2015). Prevalence of Cancer in Patients with Thyroid Nodules in the Island of Cyprus: Predictive Value of Ultrasound Features and Thyroid Autoimmune Status. Eur. Thyroid. J..

[41] Papaioannou C., Lamnisos D., Kyriacou K., Lyssiotis T., Constantinides V., Frangos S., Economides A., Economides P.A. (2020). Lymph Node Metastasis and Extrathyroidal Extension in Papillary Thyroid Microcarcinoma in Cyprus: Suspicious Subcentimeter Nodules Should Undergo FNA When Multifocality Is Suspected. J. Thyroid. Res..

[42] Haugen B.R., Alexander E.K., Bible K.C., Doherty G.M., Mandel S.J., Nikiforov Y.E., Pacini F., Randolph G.W., Sawka A.M., Schlumberger M. (2016). 2015 American Thyroid Association Management Guidelines for Adult Patients with Thyroid Nodules and Differentiated Thyroid Cancer: The American Thyroid Association Guidelines Task Force on Thyroid Nodules and Differentiated Thyroid Cancer. Thyroid.

[43] Cross P., Chandra A., Giles T., Johnson S., Kocjan G., Poller D., Stephenson T. (2016). Guidance on the Reporting of Thyroid Cytology Specimens.

[44] Al Mohareb O., Al Saqaaby M., Ekhzaimy A., Hamza M., AlMalki M.H., Bamehriz F., Abukhater M., Brema I. (2021). The Relationship Between Thyroid Function and Body Composition, Leptin, Adiponectin, and Insulin Sensitivity in Morbidly Obese Euthyroid Subjects Compared to Non-Obese Subjects. Clin. Med. Insights Endocrinol. Diabetes.

[45] Zynat J., Li S., Ma Y., Han L., Ma F., Zhang Y., Xing B., Wang X., Guo Y. (2020). Impact of Abdominal Obesity on Thyroid Auto-Antibody Positivity: Abdominal Obesity Can Enhance the Risk of Thyroid Autoimmunity in Men. Int. J. Endocrinol..

[46] Rotondi M., Magri F., Chiovato L. (2011). Thyroid and Obesity: Not a One-Way Interaction. J. Clin. Endocrinol. Metab..

[47] Yao J.Y., Liu P., Zhang W., Wang K.W., Lyu C.P., Zhang Z.W., Chen X.L., Chen Y., Wang X.S., Ding Y.X. (2021). Obesity Rather than Metabolic Syndrome Is a Risk Factor for Subclinical Hypothyroidism and Thyroid Autoimmunity. Biomed. Environ. Sci..

[48] Baranowska-Bik A., Bik W. (2020). The Association of Obesity with Autoimmune Thyroiditis and Thyroid Function-Possible Mechanisms of Bilateral Interaction. Int. J. Endocrinol..

[49] Valea A., Carsote M., Moldovan C., Georgescu C. (2018). Chronic Autoimmune Thyroiditis and Obesity. Arch. Balk. Med. Union.

[50] García-García E., Vázquez-López M.A., García-Fuentes E., Galera-Martínez R., Gutiérrez-Repiso C., García-Escobar I., Bonillo-Perales A. (2016). Thyroid Function and Thyroid Autoimmunity in Relation to Weight Status and Cardiovascular Risk Factors in Children and Adolescents: A Population-Based Study. JCRPE J. Clin. Res. Pediatr. Endocrinol..

[51] González-Mereles A.P., Arguinzoniz-Valenzuela S.L., López-López A.P., Maquedatenorio S.E., González-Baqué I. (2021). Overweight and Obesity in Children and Adolescents with Chronic Autoimmune Thyroiditis. Bol. Med. Hosp. Infant. Mex..

[52] Yang H.X., Zhong Y., Lv W.H., Zhang F., Yu H. (2019). Association of Adiposity with Thyroid Nodules: A Cross-Sectional Study of a Healthy Population in Beijing, China. BMC Endocr. Disord..

[53] Lai X., Zhang B., Wang Y., Jiang Y., Li J., Gao L., Wang Y. (2019). Adiposity and the Risk of Thyroid Nodules with a High-Suspicion Sonographic Pattern: A Large Cross-Sectional Epidemiological Study. J. Thorac. Dis..

[54] Xu L., Zeng F., Wang Y., Bai Y., Shan X., Kong L. (2021). Prevalence and Associated Metabolic Factors for Thyroid Nodules: A Cross-Sectional Study in Southwest of China with More than 120 Thousand Populations. BMC Endocr. Disord..

[55] Hu L., Li T., Yin X.L., Zou Y. (2020). An Analysis of the Correlation between Thyroid Nodules and Metabolic Syndrome. Endocr. Connect..

[56] Moon J.H., Hyun M.K., Lee J.Y., Shim J.I., Kim T.H., Choi H.S., Ahn H.Y., Kim K.W., Park D.J., Park Y.J. (2018). Prevalence of Thyroid Nodules and Their Associated Clinical Parameters: A Large-Scale, Multicenter-Based Health Checkup Study. Korean J. Intern. Med..

[57] Chen Y., Zhu C., Chen Y., Wang N., Li Q., Han B., Zhao L., Chen C., Zhai H., Lu Y. (2018). The Association of Thyroid Nodules with Metabolic Status: A Cross-Sectional SPECT-China Study. Int. J. Endocrinol..

[58] de Siqueira R.A., dos Santos Rodrigues A.P., Miamae L.M., Tomimori E.K., Silveira E.A. (2019). Thyroid Nodules in Severely Obese Patients: Frequency and Risk of Malignancy on Ultrasonography. Endocr. Res..

[59] Zhao S., Jia X., Fan X., Zhao L., Pang P., Wang Y., Luo Y., Wang F., Yang G., Wang X. (2019). Association of Obesity with the Clinicopathological Features of Thyroid Cancer in a Large, Operative Population: A Retrospective Case-Control Study. Medicine.

[60] Lai X., Xia Y., Zhang B., Li J., Jiang Y. (2017). A Meta-Analysis of Hashimoto’s Thyroiditis and Papillary Thyroid Carcinoma Risk. Oncotarget.

[61] Abbasgholizadeh P., Naseri A., Nasiri E., Sadra V. (2021). Is Hashimoto Thyroiditis Associated with Increasing Risk of Thyroid Malignancies? A Systematic Review and Meta-Analysis. Thyroid Res..

[62] Ferrari S.M., Fallahi P., Elia G., Ragusa F., Ruffilli I., Paparo S.R., Antonelli A. (2020). Thyroid Autoimmune Disorders and Cancer. Semin. Cancer Biol..

[63] Zosin I., Balaş M. (2013). Clinical, Ultrasonographical and Histopathological Aspects in Hashimoto’s Thyroiditis Associated with Malignant and Benign Thyroid Nodules. Endokrynol. Pol..

[64] Peterson E., De P., Nuttall R. (2012). BMI, Diet and Female Reproductive Factors as Risks for Thyroid Cancer: A Systematic Review. PLoS ONE.

[65] Son H., Lee H., Kang K., Lee I. (2018). The Risk of Thyroid Cancer and Obesity: A Nationwide Population-Based Study Using the Korea National Health Insurance Corporation Cohort Database. Surg. Oncol..

[66] Jankovic B., Le K.T., Hershman J.M. (2013). Hashimoto’s Thyroiditis and Papillary Thyroid Carcinoma: Is There a Correlation?. J. Clin. Endocrinol. Metab..

[67] Shi L., Zhou L., Wang J., Li F., Xie L. (2017). Cytokine Production of Papillary Thyroid Carcinoma Coexisting with Hashimoto’s Thyroiditis. Int. J. Clin. Exp. Pathol..

[68] Zivancevic-Simonovic S., Mihaljevic O., Majstorovic I., Popovic S., Markovic S., Milosevic-Djordjevic O., Jovanovic Z., Mijatovic-Teodorovic L., Mihajlovic D., Colic M. (2015). Cytokine Production in Patients with Papillary Thyroid Cancer and Associated Autoimmune Hashimoto Thyroiditis. Cancer Immunol. Immunother..

